# A Conceptual Framework for Integrating Cellular Protein Folding, Misfolding and Aggregation

**DOI:** 10.3390/life11070605

**Published:** 2021-06-24

**Authors:** Seong Il Choi, Baik L. Seong

**Affiliations:** 1 Department of Biochemistry and Biophysics, Stockholm University, SE 106 91 Stockholm, Sweden; 2 Department of Biotechnology, College of Life Science and Biotechnology, Yonsei University, Seoul 03722, Korea; 3 Vaccine Innovation Technology Alliance (VITAL)-Korea, Yonsei University, Seoul 03722, Korea

**Keywords:** protein folding, misfolding, aggregation, chaperones, intermolecular repulsions, excluded volume, surface charges

## Abstract

How proteins properly fold and maintain solubility at the risk of misfolding and aggregation in the cellular environments still remains largely unknown. Aggregation has been traditionally treated as a consequence of protein folding (or misfolding). Notably, however, aggregation can be generally inhibited by affecting the intermolecular interactions leading to aggregation, independently of protein folding and conformation. We here point out that rigorous distinction between protein folding and aggregation as two independent processes is necessary to reconcile and underlie all observations regarding the combined cellular protein folding and aggregation. So far, the direct attractive interactions (e.g., hydrophobic interactions) between cellular macromolecules including chaperones and interacting polypeptides have been widely believed to mainly stabilize polypeptides against aggregation. However, the intermolecular repulsions by large excluded volume and surface charges of cellular macromolecules can play a key role in stabilizing their physically connected polypeptides against aggregation, irrespective of the connection types and induced conformational changes, underlying the generic intrinsic chaperone activity of cellular macromolecules. Such rigorous distinction and intermolecular repulsive force-driven aggregation inhibition by cellular macromolecules could give new insights into understanding the complex cellular protein landscapes that remain uncharted.

## 1. Introduction

Cellular proteins navigate diverse conformational landscapes including folding, misfolding and aggregation in the cellular environments where polypeptides are physically connected to (or surrounded by) crowded cellular macromolecules with large excluded volume and attractive/repulsive surface area [1,2,3,4]. Such landscapes seem to be possibly explained in terms of protein conformation. For example, aggregation with an irreversible tendency has been traditionally viewed as a consequence of misfolding [5]. Consistently, the aggregation-associated neurodegenerative diseases including Alzheimer’s disease and Parkinson’s disease are commonly called conformational disorder (misfolding or protein folding) diseases [6,7]. Protein’s biological functions including specific enzymatic functions and binding are strictly dependent on protein conformation, highlighting the importance of the structural information of native proteins as well as aggregates. Similarly, the aggregation (or solubility) properties of proteins can be greatly different, depending on their conformations such as unfolded, misfolded and folded states. Protein conformation, together with folding rate and thermodynamic stability, are therefore the important determinants for aggregation. Accordingly, the effects of intrinsic and extrinsic factors on the protein-folding properties in terms of kinetics, thermodynamics and structure have been widely believed to underlie their corresponding effects on aggregation as pointed out previously [8]. However, this prevailing view should be carefully applied to aggregation with the caveats, as will be manifested throughout this paper. Otherwise it could be misleading in understanding, in particular, the effects of cellular macromolecules including molecular chaperones on the combined cellular protein folding and aggregation.

**Figure 1:**
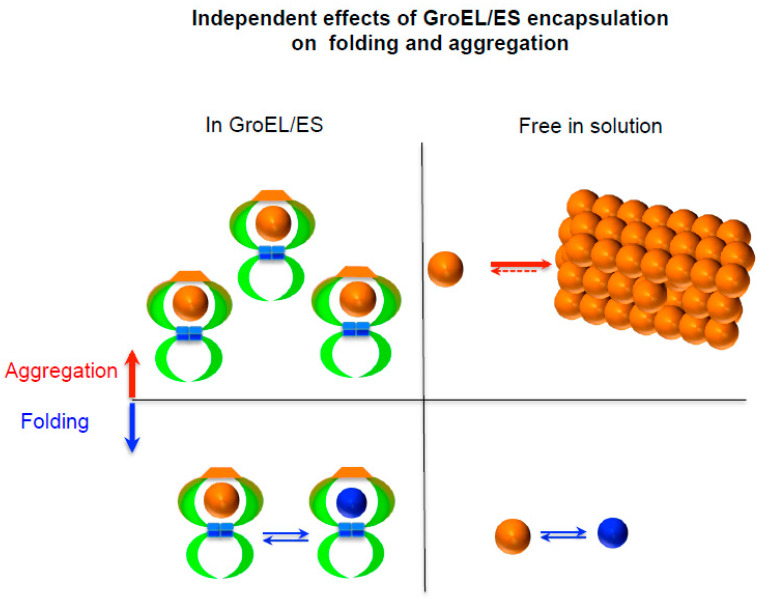
Independency between protein folding and aggregation in the chamber of GroEL/ES. A client protein in the chamber of GroEL/ES is completely protected from the self-aggregation by the encapsulation, independently of protein folding and client’s conformations. The encapsulation independently affects protein folding and aggregation. Orange and blue spheres represent unfolded and folded states, respectively.

In contrast to the prevailing view, however, the intermolecular attractive interactions between proteins leading to aggregation can be generally inhibited by the intrinsic and extrinsic factors, independently of protein folding and conformation. For example, charges are the major determinants dictating solubility or aggregation inhibition of molecules including chemicals, cellular macromolecules and colloids in the aqueous phase due to intermolecular repulsions including direct repulsions and desolvation penalty [9,10]. The surface charges of proteins are crucial for solubility maintenance or aggregation inhibition whereas they can be dispensible for protein folding and thermodynamic stability [11]. Proteins can be more aggregation-resistant by the surface charge engineering even with sacrificing thermodynamic stability [12]. Moreover, chaperones can prevent aggregation of their substrate by shielding aggregation-prone regions through direct attractive interactions (e.g., hydrophobic interactions) or steric masking (e.g., encapsulation of GroEL/ES) [13]. Protein in the chamber of GroEL/ES is protected from aggregation, independently of its conformations; the encapsulation independently affects protein folding and aggregation as shown in Figure 1. Remarkably, the cellular macromolecules including chaperones were suggested to generally exhibit the intrinsic chaperone activity to prevent the aggregation of their physically connected polypeptides irrespective of the connection types between them, due to the intermolecular repulsions resulting from their large excluded volume and surface charges [14,15,16,17]. Such intermolecular repulsion-driven aggregation inhibition (named ‘a social distancing measure’), distinct from conformational changes and direct attractive interactions, can play a key role in solubility maintenance of proteins against aggregation at the whole proteome level [4]. Consistently, protein charges at the proteome level in bacterial species were suggested to have evolved to maintain protein solubility against aggregation by the electrostatic repulsions in the crowded cellular conditions [18]. Notably, the destabilizing forces by the intrinsic and extrinsic factors against aggregation can be unrelated to protein folding and conformation, in contrary to the favorable attractive interactions for aggregation, attributed to protein conformation. A new conceptual framework for describing the protein folding property-dependent and -independent aggregation is necessary. A simplified model that rigorously distinguishes protein folding and aggregation as the two independent processes was shown to conceptually reconcile and underlie all observations regarding the combined protein folding and aggregation in any given conditions without conceptual conflict [8].

The hydrophobic interactions between chaperones and their substrates have been known to be important for the substrate recognition and binding [19,20]. These interactions allow chaperones to perform the diverse protein-quality control functions. Moreover, these interactions have been widely believed to mainly stabilize the substrate proteins against aggregation. In contrast to this generalization, the quantitative analysis of the forces of cellular macromolecules including chaperones that stabilize their physically connected polypeptides against aggregation remains a formidable task due to the inherent complexity. Aggregation can be driven by hydrogen bonds or electrostatic interactions. Chaperones can recognize their substrates mainly by electrostatic interactions [21,22]. Calnexin and calreticulin chaperones bind to the glycan moieties of substrate proteins [23]. The common major stabilizing factors of chaperones and chaperone-like cellular macromolecules were proposed to be the intermolecular repulsions resulting from their excluded volume and surface charges [15,24]. Consistently, the major stabilizing factors for the colloidal stability against aggregation have been known to the intermolecular repulsions from their surface charges and excluded volume of polymers attached to the colloid surfaces [25,26]. Attractive interactions are the driving forces for the structural formation of folded proteins, aggregates and native assemblies, as well as binding between molecules. Thus, the current knowledge of protein science is largely based on intramolecular and intermolecular attractive interactions. Consistently, the protein folding and aggregation energy landscapes are described as a function of conformation driven by the stabilizing attractive interactions found in the final structures together with conformational entropy as a destabilizing factor [2,27,28,29]. In contrast, the destabilizing intermolecular steric and electrostatic repulsions against molecular compaction are difficult to recognize in the final structures [8,30]. Moreover, these forces that are challenging to elucidate are not the issues of researchers in structural biology, protein folding, aggregation based on protein conformation, binding and chaperone field. As a consequence, the destabilizing intermolecular repulsive forces of cellular macromolecules against molecular compaction have been largely ignored in describing the cellular protein landscapes. It is noteworthy that the prevailing view based on protein folding and conformation, the direct attractive interaction-mediated aggregation inhibition and the energy landscape theory can be almost blind to the intermolecular repulsion-driven aggregation inhibition generally underlying the solubility maintenance of molecules including proteins against aggregation. Solubility maintenance of molecules can be achieved when the destabilizing forces are dominant over the stabilizing attractive interactions favoring the association between molecules. In reality, each landscape of protein folding and aggregation is therefore shaped by a balance between all favorable (e.g., stabilizing attractive interactions) and unfavorable (e.g., intermolecular repulsions, non-native attractive interactions and conformational entropy) forces including constraints in given conditions.

We here point out the necessity of rigorous distinction between protein folding (or misfolding) and aggregation as two independent processes for properly integrating protein folding, misfolding and aggregation and the potential importance of the intermolecular repulsive force-driven aggregation inhibition underlying the generic intrinsic chaperone activity of cellular macromolecules including chaperones.

## 2. Relationship between Protein Folding and Aggregation

In contrast to the prevailing view treating aggregation as a consequence of protein folding (or misfolding), they need to be rigorously distinguished as two independent processes to properly integrate them and to underlie the effects of the intrinsic and extrinsic factors on the combined protein folding and aggregation without conceptual conflict or misunderstanding.

### 2.1. Independency between Protein Folding and Aggregation

The protein folding properties and the direct attractive interactions between proteins and cellular macromolecules can be described with a single protein molecule whereas aggregation is difficult to explain or predict with the behaviors of a single molecule. The relationship between protein folding and aggregation can be well illustrated and visualized using a single protein in the GroEL/ES chamber (Figure 1). In the chaperone field, the protein folding in this chamber has been the subject of interest. (i) Protein folding in the chamber can be accelerated by the confinement reducing conformational freedom [31]. (ii) The chamber can act as unfoldase (or destabilization) to resolve the kinetically trapped intermediate, giving a chance for protein to refold [32]. (iii) Protein folding occurs such as free in solution [33]. This chamber (or encapsulation by steric masking) was called the Anfinsen cage that can mimic the infinite dilution free of aggregation [34]. Notably, the protein in the chamber is completely safe from the self-aggregation regardless of (or independently of) its conformations and protein folding. It is obvious that protein folding and aggregation in the chamber are the two independent processes. The aggregation landscape in the chamber is dictated by the encapsulation itself, independently of protein conformation. Aggregation inhibition by the encapsulation or steric masking cannot be explained with protein folding, conformational entropy and the direct attractive interactions between a client protein and wall surface of the chamber and vice versa because they are independent. Notably, the encapsulation as one extrinsic factor independently and simultaneously affects both protein folding and aggregation landscapes. This conclusion can be generally applicable to any intrinsic and extrinsic factors as further described below. Due to this independency, inherently, aggregation cannot be explained with protein folding (or misfolding) consistently and sufficiently and vice versa; paradoxically, they can therefore be integrated without any conceptual conflict. There are many outstanding protein-folding models including the Anfinsen’s thermodynamic hypothesis, in vitro and in vivo folding of small proteins, energy landscape theory, cotranslational folding, binding-coupled protein folding and molecular chaperone concept. They need to be applied to aggregation with a constraint of the independency between protein folding and aggregation, as exemplified in the GroEL/ES chamber.

Such independency can be further demonstrated. The importance of the factors to stabilize proteins against aggregation was mentioned in the Introduction. When these factors are considered only for one fixed state among unfolded, misfolded and folded states of proteins, the factors are not related to the intramolecular conformational change between them at all. Protein folding and conformational change cannot address why, for example, unfolded (misfolded or folded) conformers maintain their solubility against aggregation in given environments. Indeed, the unfolded proteins maintain their solubility mainly by their charged (or hydrophilic) moieties. Similarly, the solubility maintenance and aggregation (e.g., precipitation or crystallization) of folded proteins are independent of protein folding. Moreover, aggregation monomers are generally treated as hard-spheres or structurally invariants in aggregation studies [35]. In this model, the effects of the extrinsic factors (e.g., chaperones) on aggregation pathways and structures cannot be explained by their effects on protein folding properties. Aggregation is a complex process including multiple, different steps and heterogeneous aggregates [36]. Each step can be independently controlled. Taken together, aggregation is not a simple consequence of protein folding or misfolding; they can be independent although closely related to each other due to the great difference in the aggregation behavior between the intramolecular conformers. Conversely, if there is little or no difference in such behavior, protein folding and intramolecular conformations have little or no effect on aggregation.

The necessity of the rigorous distinction between protein folding (or misfolding) and aggregation as two independent processes can be further illustrated in the case of Janus faced molecular chaperones, the core molecules at the interfaces of protein folding, misfolding and aggregation. Cellular proteins can continuously undergo the kinetic/thermodynamic partitioning between protein folding and aggregation even before folding [8,13]. Productive protein folding (or de novo protein folding) yields are largely affected by aggregation. In general, chaperones are described to ‘assist’ protein folding by preventing misfolding and aggregation in most literatures. In some cases, chaperones are reported to accelerate protein-folding rate by avoiding or overcoming the kinetic traps or misfolding (‘direct folding’ assistance) [31,37,38,39]. In most cases, however, chaperones inhibit protein folding and thermodynamically destabilize their substrates in the absence of aggregation whereas they increase the productive folding yields by preventing aggregation (‘indirect’ folding assistance) [40,41,42,43,44,45]. The indirect folding assistance is frequently misunderstood as the direct folding assistance in the protein science community. The folding assistance by prevention of aggregation with the antifolding activity and thermodynamic destabilization appears to be a self-contradictory concept in terms of the classic intramolecular protein folding. This conundrum is difficult to solve solely with the protein folding properties. Indeed, the rescue of a misfolded monomer in the intramolecular reaction is independent of the prevention of intermolecular association between misfolded monomers. The seemingly self-contradictory concept and widespread misunderstanding of chaperones can be definitely resolved when the chaperone’s effects on protein folding (or misfolding) is rigorously distinguished from their effects on aggregation, consistent with the aforementioned independency between protein folding and aggregation in the chamber of GroEL/ES. Moreover, the application of such distinction to the intramolecular and intermolecular reactions mediated by chaperones could be helpful for understanding the versatile chaperone functions including unfolding/folding, antiaggregation (or disaggregation) /aggregation, disassembly/assembly and protein trafficking requiring antifolding. 

The Anfinsen’s thermodynamic hypothesis is a tenet of protein folding that the native structures of proteins are thermodynamically most stable under the physiological conditions and thus fold spontaneously [46]. The relationship between The Anfinsen’s thermodynamic hypothesis and aggregation can be explained with the aforementioned relationship between protein folding and aggregation. The Anfinsen’s thermodynamic hypothesis is strictly limited to the intramolecular conformation change between the unfolded and folded states in a given condition; aggregation should be excluded in the protein folding thermodynamics. Intramolecular forces cannot represent intermolecular forces and vice versa because they are independent. Even folded proteins with high thermodynamic stability should be additionally decorated on their surfaces with the optimum charges in order to maintain their solubility. High thermodynamic stability alone cannot ensure safety from aggregation [47]. Thus, molecular chaperone concept introduced the assisted protein folding instead of the spontaneous protein folding [3,13]. Moreover, native protein structures were suggested to be thermodynamically unstable towards amyloid fibrils or non-amyloidogenic aggregates in the combined protein folding and aggregation landscape, indicating that proteins are potentially vulnerable to aggregation [48,49,50,51]. The metastability was suggested to be involved in the aggregation-associated neurodegenerative diseases [52]. Thus, it is of great importance in protein science and biomedicine to understand how proteins overcome this metastability in terms of kinetics and thermodynamics in the cellular environments. These kinetic and thermodynamic aggregation problems are inherently beyond the Anfinsen’s thermodynamic hypothesis and protein folding. In addition to proper protein folding and thermodynamic stability, the surface charges of proteins, chaperone assistance and the physical connection of proteins with cellular macromolecules are necessary to combat aggregation in the cellular environments [8]. Chaperones are essential for aggregation control whereas they can be dispensible for protein folding. The chaperone-assisted protein folding by preventing aggregation and the aforementioned metastability raise a fundamental question of whether they challenge the Anfinsen’s thermodynamic hypothesis. Due to the independency between protein folding and aggregation, however, these aggregation problems do not challenge the validity of the Anfinsen’s thermodynamic hypothesis and the protein folding principles. Anfinsen knew that aggregation decreased the refolding yields in his experiments [53]. To our knowledge, confusion or misunderstanding of intramolecular thermodynamic system with intermolecular thermodynamic system has not been found in the cases of other molecules except for proteins. In order to properly integrate the Anfinsen’s thermodynamic hypothesis including the protein folding principles with aggregation, we should first distinguish between the intramolecular protein folding and intermolecular aggregation as the two independent thermodynamic systems. In this paper, protein folding is described as the intramolecular compaction within a monomer to compare the intermolecular compaction or aggregation between the monomers. Even though the native forms of proteins can consist of quaternary structures, the independency between protein folding and aggregation is valid. 

### 2.2. A Conceptual Framework for Integrating Protein Folding, Misfolding and Aggregation

**Figure 2:**
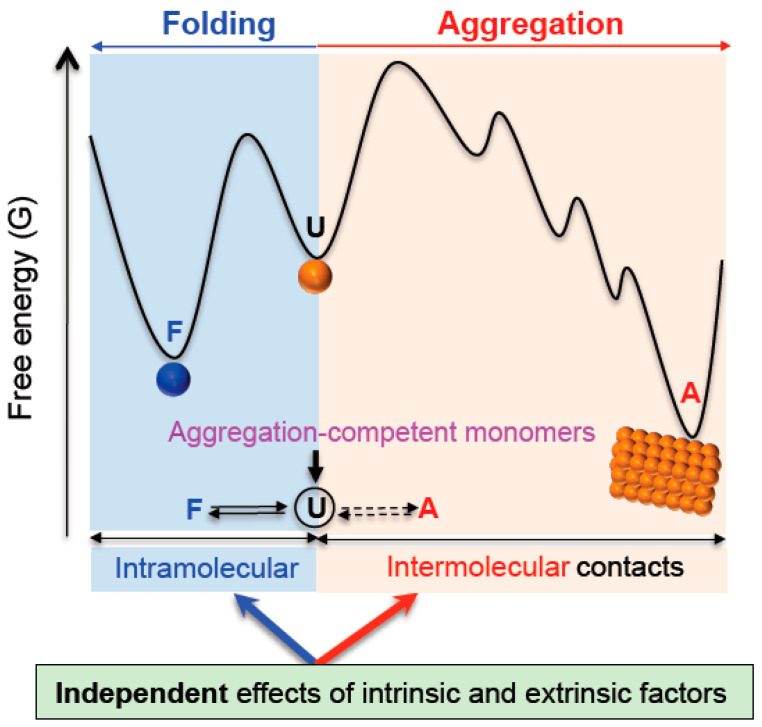
A unifying model for integrating protein folding and aggregation. Protein folding and aggregation free energy landscapes are independent (see the text for detailed description). The two landscapes are overlapped with the aggregation-competent monomers (or common monomers). Due to their independency, the intrinsic and extrinsic factors independently affect them as indicated by arrows. F, U, and A represent folded, unfolded and aggregated states, respectively. This figure is adapted from [8].

When protein folding and aggregation are treated as the two independent, combined intramolecular and intermolecular reactions (Figure 2), this model was shown to conceptually underlie all observations including the protein folding property-independent and -dependent aggregation and the indirect assistance of chaperones, as previously described [8]. The kinetic and thermodynamic parameters of each elementary step of the combined intramolecular and intermolecular reactions of molecules are independent. The parameters of protein folding (e.g., equilibrium constants and folding/unfolding rate constants) are independent of the parameters of aggregation (e.g., binding equilibrium constants and association/dissociation rate constants). As a consequence, the protein folding and aggregation free energy landscapes that are described with these parameters are independent. This principle underlies the independency between protein folding and aggregation we focus on in this paper. These two free energy landscapes can be combined together or communicated through ‘the aggregation-competent monomers’ (the common monomers that are overlapped between the two landscapes). The concept of the common monomers can be seen in describing the combined protein folding and aggregation energy landscape [2]. The common monomers can be unfolded, misfolded, intermediates and folded conformers. Depending on the conformation of the common monomers, the aggregation free energy landscape can be greatly different in the case of proteins. Moreover, thermodynamic stability determines the concentration of the common monomers in a given total protein concentration and environment. At the resulting concentration of the common monomers, aggregation can be described by the aggregation free energy landscape. This description reconciles and underlies the protein folding property-dependent and -independent aggregation without any conceptual conflict that otherwise would be seemingly contradictory. Importantly, due to the independency of the protein folding and aggregation free energy landscapes, the intrinsic and extrinsic factors ‘independently’ affect both landscapes in terms of kinetics, thermodynamics and structure, consistent with the chamber of GroEL/ES. Such factors include the surface properties of aggregation monomers, conformational entropy, attractive/repulsive interactions, cellular macromolecules, native complexation, ions, temperature, osmolytes, pH, detergents and so on. Chaperones, surface charges and conformational entropy can destabilize (or inhibit protein folding) proteins thermodynamically, increasing the concentration of the common monomers and thus potential aggregation tendency. Independently, however, they can destabilize aggregation at the same time. Aggregation is affected by their independent effects on both protein folding and aggregation free energy landscapes. This is a reason why, for example, the effect of conformational entropy on aggregation cannot be explained accurately and consistently with its effect on protein folding. This model also clarifies that the aggregation inhibition with sacrificing protein folding rate and thermodynamic stability and the protein conformation- independent aggregation inhibition does not challenge the protein folding property-dependent aggregation description. Consistent with the GroEL/ES chamber, this model rationalizes why cellular macromolecules can generally reshape the aggregation free energy landscape of their physically connected polypeptides by their intermolecular repulsive forces as well as the intermolecular attractive interactions, independently of their corresponding effect on the protein folding free energy landscape [4,8].

## 3. A Social Distancing Measure Underlying the Generic Intrinsic Chaperone Activity of Cellular Macromolecules

The magnitude of steric and electrostatic repulsions can overwhelm that of stabilizing attractive interactions for biomolecular assembly and binding. The excluded volume of molecules is a constraint for all biomolecular assembly and binding; for example, the excluded volume of a single hydrogen atom cannot be violated. Conformational space violating excluded volume cannot be accessible to proteins [30]. Consistently, a significant fraction of rotational φ and ψ angles around peptide bond do not exist due to steric clash [30,54]. When proteins cannot avoid the excluded volume repulsions, they can be denatured, for example, in the narrow channels of translocons, proteasomes and ribosomes. Similarly, it was reported that only one domain of two domains in a multidomain protein can fold at the same time due to the excluded volume repulsions of the two folded domains in too close spatial proximity [55]. It should be noted that the direct (or original) excluded volume repulsions against molecular compaction we here focus on is basically different from the indirect stabilizing effect of the excluded volume repulsions as follows. Reduced volume available to proteins due to the excluded volume (30–40% volume occupancy) of crowded cellular macromolecules can thermodynamically favor more compact states including folded and aggregated structures [56]. So far, the cellular protein landscapes have been largely discussed with this indirect stabilizing excluded volume effect by macromolecular crowding that does not include the physical connection of cellular macromolecules with proteins.

Transfer a charged residue from water to hydrophobic core can generate a large desolvation penalty up to 19 kcal/mol [57,58]. Considering that protein stability ranges from 2 to 10 kcal/mol, protein folding occurs in a constraint of this desolvation penalty together with the excluded volume. Likewise, the surface charges of peptides can inhibit the intermolecular association leading to aggregation. Consistently, the net charge of proteins is crucial for their solubility against aggregation; solubility is lowest at isoelectric point [59,60]. Mutations in the charged residues decreasing the intermolecular electrostatic repulsions are involved in the aggregation-associated diseases [61]. Similar to the conventional colloid stability, the solubility maintenance of cellular macromolecules can be dictated by the intermolecular electrostatic repulsions [18].

**Figure 3:**
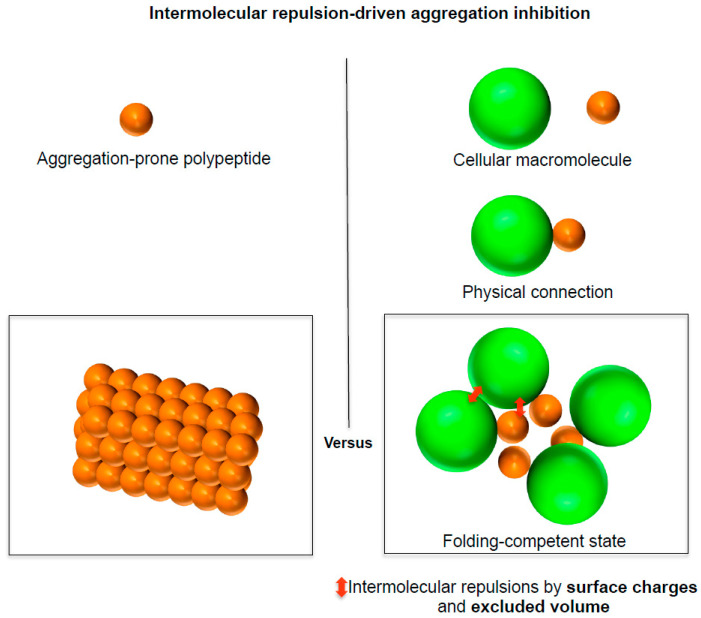
Intermolecular repulsion-driven aggregation inhibition by cellular macromolecules. The intermolecular repulsions (red arrows) by large excluded volume and surface charges of cellular macromolecules (green spheres) inhibit the aggregation of the physically connected polypeptides (orange sphere) regardless of the physical connection type, keeping the polypeptide in a folding-competent state. This figure is adapted from [4].

The following reasons further support the intermolecular repulsive force-driven aggregation inhibition by cellular macromolecules. Aggregation is a multimolecular assembly of polypeptides that brings involved molecules in close proximity to each other increasing the repulsions between molecules; aggregation is neither unimolecular nor bimolecular reaction. Furthermore, it can be specific [62,63]. Aggregation is largely affected by the surface properties of aggregation monomers. The large excluded volume of bulky cellular macromolecules cannot be violated during the aggregation of their physically connected polypeptides. Moreover, polypeptides physically interact with different cellular macromolecules at the same time in the cellular environments. Cellular macromolecules are polyions with many surface charges and huge excluded volume relative to a single charged residue. As illustrated in Figure 3, the physical connection of cellular macromolecules to the surface of aggregation-prone polypeptides can therefore inhibit the aggregation of the polypeptides irrespective of the physical connection types and resulting conformational changes [4,8,24]. The complexed structures between cellular macromolecules and polypeptides can be treated as a single molecule. Thus, their physical behaviors such as diffusion and tumbling can be synchronized in the linkage context; they can be greatly influenced by the bulky cellular macromolecules. Indeed, this principle is used for measuring the quinary interactions (or transient interactions) between crowded cellular macromolecules and proteins of interest using NMR [64]. Similar to the protein movement landscape, aggregation landscapes of proteins can be reshaped upon the physical connection of bulky cellular macromolecules (e.g., encapsulation by GroEL/ES and ribosome tethering).

The intermolecular repulsions exhibit the following unique features, underlying the generic intrinsic chaperone activity of cellular macromolecules as mentioned previously [8]. First, they automatically generate upon the physical connection of cellular macromolecules with polypeptides regardless of the connection types and induced conformational changes. Second, the magnitude of them can be increased depending on the size and surface area of molecules. Third, they can inhibit aggregation without the direct attractive interactions between cellular macromolecules and the aggregation-prone regions of the connected polypeptides. This far long-range effect was called allosteric effect of cellular macromolecules in terms of aggregation [17]. Fourth, they can inhibit aggregation irrespective of the nature of aggregation stabilizing forces (e.g., hydrophobic interactions and non-hydrophobic interactions).

### 3.1. A Social Distancing Measure by Chaperones

The intermolecular repulsive forces by excluded volume and surface charges can be important for the chaperone’s substrate stabilization against aggregation. Indeed, the physical entity of the encapsulation by GroEL/ES and TriC/CCT and shielding (or wrapping) of Trigger factor, SecB and Hsp90 is a steric masking resulting from their excluded volume repulsions. The entropic pulling forces of Hsp70 resulting from its excluded volume repulsions were suggested to underlie its diverse functions such as unfolding, disaggregation and translocation [65]. Similarly, the steric repulsions of DnaK (an *E. coli* Hsp70 homolog) molecules can induce the expansion of their bound substrates or unfolding, rescuing the kinetically trapped intermediates [39,66]. DnaK binds its substrate protein through the recognition of short linear peptides with 2-4 consecutive hydrophobic residues [20]. Thus, it is conceivable that most of the hydrophobic regions of substrate protein are exposed to solvent in DnaK-client complex, raising a question of whether the direct hydrophobic interactions for the substrate recognition is the major factors to stabilize the client against aggregation. Like Hsp70, chaperones partially mask the hydrophobic regions of their substrates through the direct hydrophobic interactions. The substrate-stabilization of DnaK was proposed to largely result from its steric and electrostatic repulsions; in the covalent fusion context, its substrate-binding residues and C-terminal domain can be dispensible for the substrate stabilization against aggregation [15]. The charge-rich regions on the surfaces of Hsp90 are important for its antiaggregation activity although they are not involved in the substrate binding [67]. The charge-rich patch of Hsp90 can modulate the aggregation without the direct binding of aggregation-prone regions of its substrate protein, consistent with the unique properties of the intermolecular repulsion-driven aggregation inhibition. This illustrates that the extrinsic charges (the surface charges of Hsp90) can behave similarly to the intrinsic charges of substrate proteins in the complexed state in terms of aggregation. The direct hydrophobic interactions between chaperones and their substrates can be insufficient for aggregation inhibition; additionally, the surfaces of chaperones should be decorated with optimum charges for aggregation inhibition. This situation is similar to the necessity of additional charge decoration on the surface of folded proteins that are stabilized by hydrophobic interactions.

### 3.2. A Social Distancing Measure by Macromolecular Tethering: A Hallmark of De Novo Protein Folding Environments

**Figure 4:**
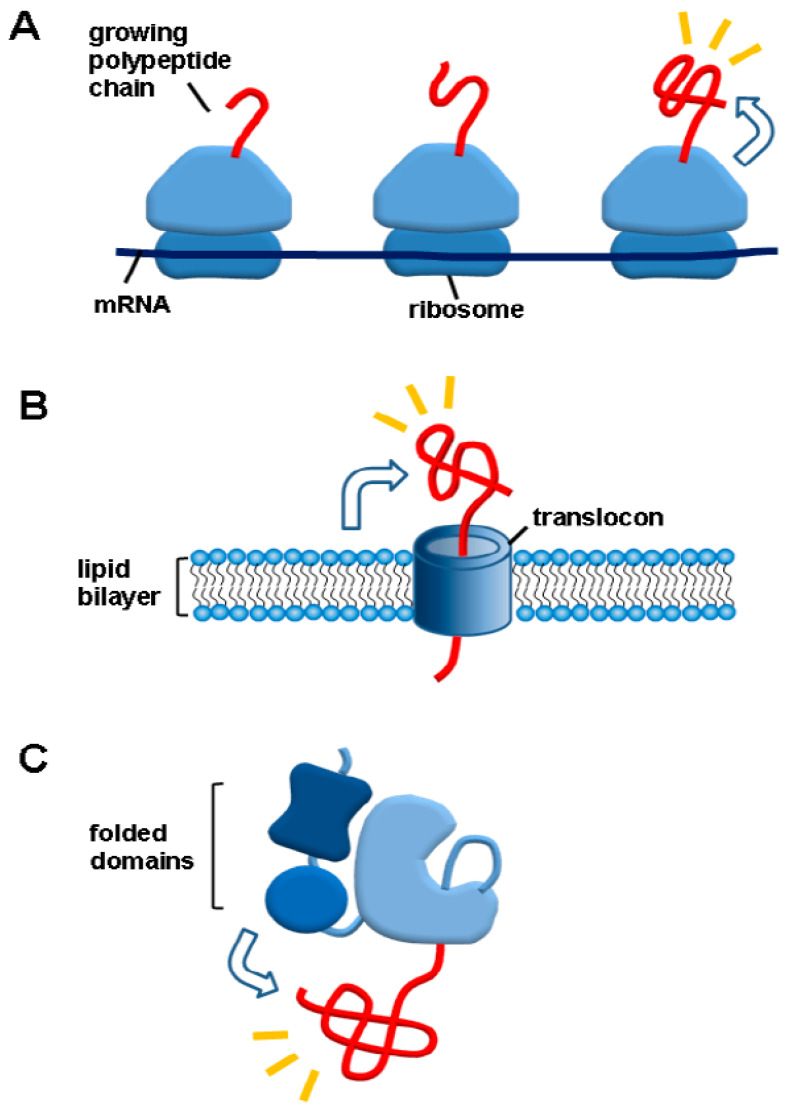
Macromolecular tethering as a hallmark of de novo protein folding environments. (**A**) Newly synthesized polypeptides are tethered to relatively gigantic ribosome with supernegative surface charges. (**B**) Approximately 30% of proteome are anchored at membranes. (**C**) More than 80% of proteome are multidomain proteins in which prefolded (cotranslationally folded) domains are tethered to unfolded domains. This figure is adapted from [24].

A hallmark of de novo protein-folding environments is the macromolecular tethering, as illustrated in Figure 4. Newly synthesized polypeptides are tethered to relatively gigantic macromolecules such as ribosomes (100% proteome), membranes (30% proteome) or (folded or unfolded domains) in case of multidomain proteins (80% proteome) [68,69]. The macromolecular tethering in cis is distinct from the reversible binding of chaperones with their substrates in trans. Ubiquitination, sumoylation and glycosylation are also kinds of the macromolecular tethering. The chaperone-assisted protein folding as well as cotranslational (or cotranslocational) folding occurs in the macromolecular tethering context. The aggregation problem of the nascent polypeptides is the subject of de novo protein folding in the chaperone field [70,71]. Nonetheless, little attention has been paid to the effect of the macromolecular tethering on the aggregation of polypeptides. In contrast, the macromolecular tethering such as ribosome display, cell surface-display and covalent tethering (or fusion) to soluble carriers have been known to be the powerful avenues to prevent aggregation and thus increase productive protein folding yields in the biotechnology field [72,73,74,75,76]. Interestingly, the artificial fusion proteins are multidomain proteins where the folded N-terminal domains (solubility-enhancing tags) exert the chaperone effect on the C-terminal aggregation-prone domains (target proteins). This cis-acting chaperone type was proposed to occur in the folding of native multidomain proteins by showing the folded N-terminal domains of the native multidomain proteins act as the powerful solubility-enhancing tags for the diverse aggregation-prone heterologous proteins [14]. Likewise, the N-terminal domain of spider silk domain that acts as a powerful solubility-enhancing tag for diverse proteins was suggested to act in the same way for its authentic C-terminal regions [77]. Ubiquitin and SUMO have been known to be powerful solubility-enhancing tags [78,79]. According to the above logic, they can exert the chaperone effect on their tagged native proteins in cis. Mechanistically, the folded N-terminal domains were suggested to keep the C-terminal domains in an aggregation-resistant and folding-competent state through the intermolecular repulsions by their excluded volume and surface charges [14]. Similar to the intermolecular repulsion-driven aggregation inhibition, intrinsically disordered polypeptides were suggested to prevent the aggregation of their connected polypeptides by their entropic bristles and hydration [80]. In this regard, gigantic ribosomes and membranes with many surface charges can inhibit the aggregation of their tethered polypeptides. Thus, cotranslational (or cotranslocational) folding of polypeptides can occur based on their sequence information in the macromolecular tethering context while aggregation of the polypeptides is prevented even without the attractive interactions between cellular macromolecules and polypeptides; this automatic helping mode was named cis-acting protein folding helper [4,8,14,81]. In contrast, the direct hydrophobic interactions between chaperones and client proteins that are crucial for the substrate recognition and binding have a tendency to inhibit protein folding; thus, binding and release cycle is necessary to promote protein folding. The weak binding between Spy chaperone and its client can allow the client protein to fold in the complexed state [82]. In this regard, the macromolecular tethering can be a good chaperone type for assisting de novo protein folding. Moreover, newly synthesized polypeptides should interact with a lot of cellular macromolecules such as peptide deformylase, methionine aminopeptidase, secretion helper proteins and glycosylating enzymes cotranslationally or cotranslocationally. The macromolecular tethering allows these interactions to occur. According to the intermolecular repulsive force-driven aggregation inhibition, it is expected that these interacting cellular macromolecules can also act as chaperones for de novo protein folding.

### 3.3. Conversion of a Protein into a Potent Chaperone: Cis/Trans Conversion

**Figure 5:**
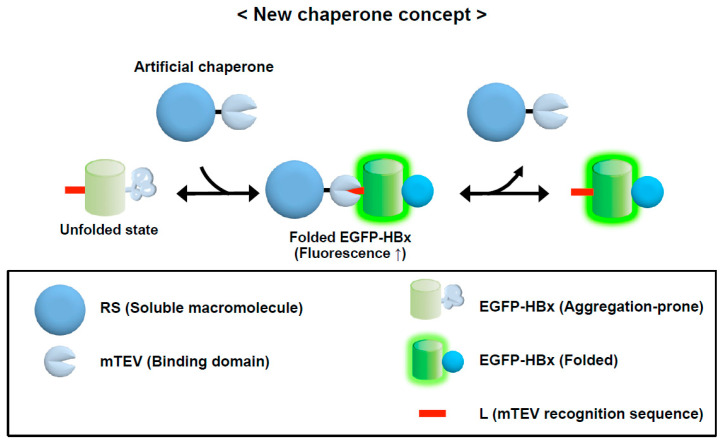
Conversion of a soluble protein into a potent chaperone. To demonstrate the intrinsic chaperone activity of cellular macromolecules, an artificial chaperone is constructed harboring a solubility-enhancing module (RS) and a substrate-recognition module (mTEV) that specifically bind to a short tag of 7 residues (L tag denoted by red bar). The aggregation-prone protein (EGFP-Hbx) with the L tag is the client protein of RS-mTEV. This figure is adapted from [17].

The intermolecular repulsion-driven aggregation inhibition and the macromolecular tethering-mediated aggregation inhibition indicate that a protein can be converted into a chaperone in trans if the physical binding between a protein and aggregation-prone protein is provided. To demonstrate this possibility, an artificial protein containing a solubility-enhancing module and an inactive protease mutant domain that can still recognize specifically a short linear sequence of 7 residues called L tag was constructed (Figure 5) [17]. This protein was shown to exhibit a potent chaperone activity for the diverse proteins with the L tag in a tag position-independent manner. It indirectly assists protein folding by preventing aggregation while the client proteins fold in the complexed state. The chaperone activity of this protein largely results from the solubility-enhancing module that is not directly involved in the substrate binding. This long-range allosteric effect is similar to the effect of the charge patch of Hsp90 on aggregation inhibition. The results of the artificial chaperone system strongly support the intrinsic chaperone activity of cellular macromolecules.

The chaperones and folding catalysts including Hsp60, Hsp70, Trigger factor, Spy, protein disulfide isomerase and peptidyl prolyl isomerase strongly prevent the aggregation of their fused proteins in cis [83,84,85,86,87]. The change of the physical connection type from trans to cis and vice versa does not affect their intrinsic chaperone activity to prevent aggregation. Diverse cellular macromolecules have been known to exhibit the moonlighting chaperone activity, including ribosomes, membranes, proteins, RNA, DNA and polyphosphate [16,88,89,90,91,92,93,94]. Taken together, the intrinsic chaperone activity of cellular macromolecules based on the intermolecular repulsions by their large excluded volume and surface charges can commonly underlie the classic chaperones, the moonlighting chaperone activity of cellular macromolecules, the macromolecular tethering as a cis-acting protein folding helper and the aforementioned cis/trans conversion as well as the surface display technology and fusion technology for aggregation inhibition.

### 3.4. Implications of a Social Distancing Measure for the Development of Therapeutic Strategies for Aggregation-Associated Diseases

The generic intrinsic chaperone activity of cellular macromolecule can be harnessed for the development of therapeutic strategies for the aggregation-associated diseases. The polypeptides involved in the diseases are physically (directly or indirectly) connected to a myriad of cellular macromolecules including domains and other proteins. Such cellular macromolecules possibly exerting the chaperone effect on their connected polypeptides can be drug targets as suggested previously [17]. Consistent with the intermolecular repulsive force-driven aggregation inhibition, small amyloid-binding molecule conjugated to a protein effectively prevents aggregation due to the protein’s steric bulkiness [95]. We here illustrate that the folding of domains and their interactions with other cellular macromolecules can modulate the aggregation of their connected flanking polypeptides. Therefore, small molecules that increase the amount (or stability) of such cellular macromolecules or the association affinity between cellular macromolecules and pathogenic polypeptides can be drug candidates. This means that the flanking domains and their interacting cellular macromolecules could be drug targets. This strategy can be especially useful due to the following reason. Many pathogenic polypeptides are the cleaved fragments or intrinsically disordered proteins that lack stable pharmacophores [96]. Antibodies against pathogenic polypeptides have been known to exhibit therapeutic potential [97]. It is largely unknown how antibodies inhibit aggregation. Antibodies are kinds of bulky cellular macromolecules. According to the intermolecular repulsion-driven aggregation inhibition, it is conceivable that the intermolecular repulsions of antibodies can stabilize the bound polypeptides against aggregation, independently of the direct attractive interactions between them and the subsequent effect on the conformational changes of the polypeptides. The intermolecular repulsive force-driven aggregation inhibition presented here suggests that the whole regions of pathogenic polypeptides including their flanking regions could be antibody targets for aggregation inhibition.

## 4. Conclusions and Perspectives

A general feature of the cellular environments is the physical connection of polypeptides with a myriad of cellular macromolecules with large excluded volume and attractive/repulsive surface area. So far, the effects of cellular macromolecules including chaperones on the combined protein folding and aggregation have been mainly understood through the lens of the protein folding properties (kinetics, thermodynamics and structure) and the direct attractive interactions between cellular macromolecules and polypeptides. In contrast to the prevailing view, we here point out that it is critical to rigorously distinguish between protein folding (or misfolding) and aggregation as two independent processes in order to properly integrate them. Due to the independency between protein folding and aggregation, the intrinsic and extrinsic factors independently affect protein folding and aggregation. Moreover, this new view rationalizes the generic intrinsic chaperone activity of cellular macromolecules by the intermolecular repulsive force-driven aggregation inhibition. The rigorous distinction between protein folding and aggregation and the intermolecular repulsive force-driven aggregation inhibition can give new insights into how proteins maintain solubility in the presence of misfolding and aggregation at the proteome level in the cellular environments.

The cellular protein landscapes in the protein science community have been mainly understood in the structure formation (including binding) and the pathway analysis of it, based on the attractive interactions stabilizing structures and binding to date. This trend can be blind to the intermolecular repulsive force-driven inhibition against the structural formation such as aggregation. For example, there is no consideration of the obvious charge effect on protein solubility in the chaperone concept and proteostasis concept that mainly handle the solubility maintenance of proteins against aggregation. Moreover, the intermolecular repulsions of megadalton-sized ribosomes with supernegative surface charges in terms of aggregation is not considered as described in the macromolecular tethering although the intermolecular repulsions of a single charged amino acid on aggregation is considered. It is rather shocking that, so far, cellular macromolecules have been mainly understood such as this ribosome case in terms of aggregation, based on protein folding and attractive interactions. The diverse cellular protein landscapes can be largely affected by the intermolecular repulsive forces of bulky cellular macromolecules physically interacting proteins. Therefore, the destabilizing intermolecular repulsive forces of cellular macromolecules, distinct (but fully compatible with) from conformational change, conformational entropy and attractive interactions, need to be considered in order to describe the diverse cellular protein landscapes consistently and accurately.

## Data Availability

No new data were created or analyzed in this study. Data sharing is not applicable to this article.
